# Earlier connectivity and specialization predict longitudinal changes in phonological and semantic specialization in 7- to 9-year-old children

**DOI:** 10.1162/IMAG.a.940

**Published:** 2025-10-17

**Authors:** Avantika Mathur, Jin Wang, James R. Booth

**Affiliations:** Department of Psychology and Human Development, Vanderbilt University, Nashville, TN, United States; School of Education and Information Studies, University of California, Los Angeles, CA, United States

**Keywords:** interactive specialization model, phonological specialization, semantic specialization, functional connectivity, longitudinal study, functional magnetic resonance imaging (fMRI)

## Abstract

A central assumption of the Interactive Specialization model (ISM) of brain development postulates that earlier interaction between brain regions (i.e., functional connectivity) drives later specialization. The goal of the current study was to examine this assumption in the language domain. Longitudinal data from 32 children at 7 and 9 years of age were analyzed. The children were given a sound judgment task and a meaning judgment task while undergoing functional magnetic resonance imaging (fMRI) to assess children’s phonological and semantic specialization, and functional connectivity in the brain. We found evidence that earlier connectivity at age 7 predicted later specialization at age 9 within the dorsal pathway for phonology and ventral pathway for semantics, therefore supporting the assumption of the Interactive Specialization model. We also found that individual differences in specialization at age 7 predicted later specialization at age 9, and this predictive effect was more pronounced for semantics than phonology. In sum, our results not only suggest a continued development of neural specialization for both phonology and semantics but also support the core hypothesis of ISM as earlier pathway connectivity within dorsal inferior frontal gyrus and posterior superior temporal gyrus appeared as a significant predictor for subsequent phonological specialization, and within ventral inferior frontal gyrus and posterior middle temporal gyrus appeared as a significant predictor for subsequent semantic specialization.

## 1 Introduction

The Interactive Specialization Model (ISM) is a theoretical framework (Johnson, 2011) that aims to explain brain development across various domains of human cognition. According to ISM, the cerebral cortex begins with broad functionality; then, over the course of development, certain regions show greater activation to specific inputs than other regions. One of the core assumptions of this model is that, during postnatal functional brain development, the cerebral cortex undergoes organization through patterns of inter-region connections. A cortical region becomes more specialized in its functional response because of its connectivity to other areas and its patterns of functional activity. Studies suggest that sophisticated language abilities emerge from specialized, but interconnected networks that undergo significant development from birth to childhood (Ghasoub et al., 2024; Xu et al., 2024). However, little research has directly examined whether the core hypothesis of ISM—earlier functional connectivity predicts later specialization—can explain the underlying neural mechanisms of language development. Addressing this question is critical because it not only helps examine models of brain development but also helps understand the driving force for language development, informing early identification and intervention of children with low language skills.

Considerable theoretical work, corroborated by empirical evidence, has delineated distinct networks within the brain that are involved in phonological and semantic processing in adults. The left superior temporal gyrus (STG) and the dorsal inferior frontal gyrus (dIFG) are implicated in phonological processing, whereas the left middle temporal gyrus (MTG) and the ventral inferior frontal gyrus (vIFG) have been linked to semantic processing (Binder et al., 2009; Friederici & Gierhan, 2013; Hickok & Poeppel, 2007). In children in the elementary school years, empirical research has also delineated specific brain areas engaged in phonological and semantic processing. Comparisons between sound and meaning-based tasks have revealed that the dIFG and posterior STG (pSTG) show increased activity during sound-related tasks, affirming their role in phonological processing, whereas the vIFG and posterior MTG (pMTG) exhibit heightened activity during meaning judgment tasks, thereby confirming their importance in semantic processing (Wang, Yamasaki, et al., 2021; Wang et al., 2023). These neuroimaging studies on children have observed a developmental progression in the specialization of phonological and semantic processing. At the age of 5–6 years, this specialization is only evident in the temporal lobe (Weiss et al., 2018). By the age of 7–8 years, the specialization has progressed to the frontal lobe, in addition to the temporal lobe (Wang, Yamasaki, et al., 2021), and by the age of 9–10 years, the patterns remain the same and the localization of language specialization is near-adult-like (Wang et al., 2023). These findings suggest a developmental increase in language specialization, consistent with the specialization hypothesis of ISM which argues that skill development is associated with increased functional specialization of the brain.

In addition to the examination of the specialization hypothesis, prior studies have tested the interactive hypothesis of ISM, which suggests that skill development is associated with efficient functional connectivity (FC). Specifically, previous studies provide evidence that FC within the language network predicts behavioral reading or language outcomes. For example, a decrease in network transitivity, which is indicative of reduced functional segregation and increased integration among reading-related brain regions, identified using a rhyming task, has been shown to predict improvement in pseudoword decoding ability in young readers (8–14 years old). Thus, consistent with ISM, the unification of the reading network through cooperative processing is a critical driver of gaining reading skills (Smith et al., 2018). Moreover, stronger rhyme-related connectivity from the dIFG to the pSTG at age 7 has been shown to predict better reading abilities at age 9, suggesting that early phonological processing proficiency supports later literacy acquisition (Wang, Pines, et al., 2021). A predictive relationship of enhanced connectivity between the pMTG and parietal brain regions with better semantic processing abilities has also been established in children aged 8–11 (Lee et al., 2016). Overall, early connectivity within phonological and semantic pathways has been associated with reading abilities at a later age, supporting the interactive hypothesis of ISM in the language domain (Lee et al., 2016; Wang, Pines, et al., 2021).

Although supportive evidence is observed for the specialization and interactive hypotheses of ISM in the language domain, very few studies have examined the core interactive specialization hypothesis of ISM, which argues that FC drives specialization. Among studies that examined different cognitive domains, only a few supported the ISM hypothesis of FC driving specialization. One study suggested that longitudinal changes in IPS connectivity occurred independently of changes in regional activation for a numerical problem-solving task and thus did not find support for the ISM hypothesis of FC driving specialization (Battista et al., 2018). Another study found that learning letter-sound relationships in an artificial orthography shapes the development of print specialization in the ventral occipitotemporal cortex of pre-reading children. This study also reported that the magnitude of this activation and the functional connectivity of the left fusiform gyrus to the parieto-occipital cortex depended on children’s learning performance, thus suggesting that specialization of the reading network emerges through the interactive tuning of different brain areas based on learning experience. However, this study did not directly test the ISM hypothesis of early functional connectivity driving later specialization (Pleisch et al., 2019). Two studies reported direct evidence of ISM that specialization can be predicted from connectivity (O’Rawe et al., 2019; Saygin et al., 2016). For instance, in O’Rawe et al. (2019), the specialization of the face and scene regions in children aged 9 to12 was observed to be influenced by their differential functional connectivity with posterior parietal regions, which likely play an important role in shaping the emergence of specialization during development from mid to late childhood. In Saygin et al. (2016), the eventual location of the visual word form area (VWFA) at age 8 was observed to be predicted from the region’s connectivity profile at age 5, an age before reading acquisition and before the region differed functionally from nearby more general shape-responsive cortex. Overall, previous studies provide inconsistent empirical evidence for the interactive specialization hypothesis of ISM. In addition, none of them focused on language specialization.

To address these literature gaps, our study examined the interactive specialization hypothesis of ISM by exploring whether early connectivity predicts later language specialization across a 2-year span using a shared longitudinal dataset on OpenNeuro.org (Wang et al., 2022; https://openneuro.org/datasets/ds003604). Prior studies have shown evidence of specialization at age 7 (Wang, Yamasaki, et al., 2021) and age 9 (Wang et al., 2023), but they have used cross-sectional data, whereas the current study employed a longitudinal design with the same participants measured at two time points (age 7 and age 9). Thus, while the overall dataset is the same, the participants differ across this study and prior studies. In this study, we selected participants from the shared longitudinal dataset who have completed both the phonological and semantic judgment tasks at both age 7 and age 9. In this pre-registered study, we used hierarchical regression models to investigate if early brain connectivity predicts later specialization for language-related neural pathways. Neurocognitive models of language have shown both functional (Friederici & Gierhan, 2013; Hickok & Poeppel, 2007; Price, 2012) and structural connections (Saur et al., 2008) between frontal and the temporal lobes. In this study, we examined functional connections using two approaches: (a) pathway analysis, defined as functional connectivity between fronto-temporal regions, and (b) network analysis, defined as functional connectivity from the targeted region to the entire language network - comprising of temporal, parietal, and frontal language regions.

We first aimed to examine if pathway connectivity between frontal and temporal regions within each linguistic process promotes its specialization. Specifically, we evaluated the functional connectivity of the frontal lobe to the temporal lobe at age 7 and addressed (a) if the connectivity between dIFG and pSTG at age 7 predicted phonological specialization at age 9 and (b) if the connectivity between vIFG and pMTG at age 7 predicted semantic specialization at age 9. Consistent with prior studies (Wang, Yamasaki, et al., 2021 and Wang et al., 2023), we directly contrasted activation elicited by an auditory sound judgment task and an auditory meaning judgment task to determine phonological and semantic specialization. We used the frontal lobe as seed regions for the primary analyses in our pre-registration because previous studies showed frontal specialization in 7–8 year olds (Wang et al., 2021), and dIFG and vIFG in the frontal lobe are relatively small anatomical structures, ensuring localization of activity.

In our primary pre-registered analyses, results showed that the temporal specialization was stronger and more stable (refer to Table 5). Thus, in our secondary pre-registered analysis we used temporal voxels as the seed. Because prior studies supporting the ISM hypothesis investigated connectivity of the targeted regions to multiple brain regions (O’Rawe et al., 2019; Saygin et al., 2016), we expanded the functional connectivity analysis from the targeted region to the entire language network - comprising of temporal, parietal, and frontal language regions. We then determined if network connectivity of (a) pSTG with the language network at age 7 predicted phonological specialization at age 9 and (b) pMTG with the language network at age 7 predicted semantic specialization at age 9. We hypothesized that if the interactive specialization hypothesis is correct, then early brain connectivity would predict later neural specialization.

## 2 Methods

### 2.1 Participants

Participants for this study were pulled from a shared data set on OpenNeuro.org (Wang et al., 2022; https://openneuro.org/datasets/ds003604). The Institutional Review Board of the University of Texas at Austin approved all the experimental procedures. Consent was collected from participants’ parents or guardians, and assent was collected from children before participation in the study. Parents or guardians were asked to complete an exclusionary survey and a developmental history questionnaire. All participants enrolled had normal hearing, normal/corrected-to-normal vision, and no learning, neurological, or psychiatric disorders. Children were asked to complete several screening tests, which included handedness questions, in which the child had to pretend to write, erase, pick up, open, and throw something, and the Diagnostic Evaluation of Language Variation (DELV) Part 1 Language Variation Status (Seymour et al., 2003). Children also completed standardized tests to assess their language skills and nonverbal IQ. General language skill was measured using the Clinical Evaluation of Language Fundamentals—Fifth Edition (CELF-5; Wiig et al., 2013). Nonverbal IQ was measured using the Kaufman Brief Intelligence Test—Second Edition (KBIT-2; Kaufman & Kaufman, 2004).

Seventy-eight participants out of 323 completed both runs of the sound and meaning tasks at age 7 and age 9. Out of the 78, 32 participants had all ‘good fMRI runs’ across both tasks. A run was considered a ‘good run’ if participants scored within an acceptable accuracy range, had no response bias on the fMRI tasks, and had acceptable levels of motion (refer to Data Analysis). A detailed excel sheet of subject selection is on Github (see link below in data and code availability).

Thus, longitudinal data from 32 (19 F, 13 M) healthy native English-speaking children at age 7 and age 9 (7.37 ± 0.32 years and 9.88 ± 0.51 years) were analyzed based on pre-registered criteria (https://doi.org/10.17605/OSF.IO/6ECYR). The 32 participants were right-handed defined as completing at least three out of the five handedness tasks with their right hand. The participants had a standardized IQ score of 80 or higher on the KBIT-2 (114 ± 14.30), and had typical language abilities, as indexed by a standardized Core Language Scale score of 80 or higher on the CELF-5 (110.06 ± 14.92) based on age 7 data. They were mainstream English speakers (9 of 15 responses for age 9 that were categorized as mainstream English on the DELV Part I Language Variation Status sub-test; two participants had a score of <9 at age 7 that changed to score >9 by age 9).

### 2.2 Experimental procedure

An event-related functional MRI design was used to measure cortical responses while participants performed an auditory phonological and semantic judgment task. In each trial, children heard two auditory stimuli presented sequentially and binaurally through earphones (Table 1). Participants were asked to judge whether the word pair they heard shared any of the same sounds during the sound task and whether the heard words were semantically related during the meaning task. They were instructed to press a “yes” or a “no” button using their right index or middle finger and to always press the “yes” button with their right index finger whenever they heard the frequency-modulated noise. They were instructed to respond to all trials as quickly and accurately as possible. For a detailed description of stimuli characteristics, stimuli selection, and experimental procedure refer to data descriptor publication (Wang et al., 2022).

**Table 1.** Examples of the stimuli in the auditory phonological and semantic judgment task.

Task	Condition	Example
Sound task	Onset (P_O)	Coat - cup
	Rhyme (P_R)	Wide - ride
	Unrelated (P_U)	Zip - cone
	Perceptual (P_C)	Shh - shh
Meaning task	Low (S_L)	Dish - plate
	High (S_H)	Dog - cat
	Unrelated (S_U)	Map - hut
	Perceptual (S_C)	Shh - shh

Note. There were four different auditory conditions for sound task: words that shared the same onset phoneme (“onset”), words that rhymed (“rhyme”), words that shared no common phonemes (“unrelated”), and a perceptual control condition that consisted of two presentations of frequency modulated noise (“perceptual”). Similarly, there were four different auditory conditions for meaning task: words that had low association (“Low”), words that had high association (“High”), words that were unrelated (“unrelated”), and a perceptual (“perceptual”) control condition that consisted of two presentations of frequency modulated noise.

#### 2.2.1 Sound task

There were four different auditory conditions for the sound task: words that shared the same onset phoneme (“Onset”), words that rhymed (“Rhyme”), words that shared no common phonemes (“Unrelated”), and a perceptual control condition that consisted of two presentations of frequency modulated noise (“Perceptual”) (Table 1). The task included a total of 96 trials divided into two separate 48-trial runs with each run containing 12 trials per condition. Within a trial, each auditory word stimulus had a duration ranging from 439 to 706 ms. The second word was presented approximately 1000 ms after the onset of the first one. Overall, in each trial, the total stimuli duration (i.e., two words with a brief pause in between) ranged from 1490 to 1865 ms and was followed by a jittered response interval that varied between 1500 and 2736 ms. A blue circle appeared simultaneously with the auditory presentation of the stimuli to help maintain attention on the task. The blue circle changed to yellow to provide a 1000 ms warning for the participants to respond, if they had not already, before moving on to the next trial. The total trial duration ranged from 3000 to 4530 ms. Each run lasted approximately 3 min.

#### 2.2.2 Meaning task

There were four different auditory conditions for the meaning task: words that had a low association (“Low”), words that had a high association (“High”), words that were unrelated (“Unrelated”), and a perceptual (“Perceptual”) control condition that consisted of two presentations of frequency modulated noise (Table 1). The task included a total of 96 trials divided into two separate 48-trial runs, with 12 trials per condition per run. Within each trial, each auditory word had a duration ranging from 500 to 700 ms. The second word was presented approximately 1000 ms after the onset of the first one. Overall, in each trial, the stimuli duration (i.e., two words with a brief pause in between) ranged from 1500 to 1865 ms and was followed by a jittered response interval between 1800 and 2701 ms. A blue circle appeared simultaneously with the auditory presentation of the stimuli to help maintain attention on the task. The blue circle changed to yellow to provide a 1000 ms warning for the participants to respond, if they had not already, before moving on to the next trial. The total trial duration ranged from 3300 to 4565 ms. Each run lasted approximately 3 min.

### 2.3 Data acquisition

All neuroimaging data were collected using a Siemens Skyra 3 T MRI scanner located at The University of Texas at Austin Imaging Research Center. All images were acquired using a 64-channel head coil. Participants were positioned supine in the MRI scanner, and foam pads were placed around the head to minimize movement. Participants were given a right-hand response box to respond to the functional imaging tasks. Visual stimuli were projected on a screen behind the scanner which participants viewed via a mirror attached to the head coil. Audio stimuli were presented through sound attenuating earphones to minimize the effects of scanner noise. During structural MRI and diffusion-weighted imaging, participants watched a movie to increase comfort. Participants were encouraged to remain still and were given breaks to talk to an experimenter between scans.

Structural MRI involved T1-weighted Magnetization Prepared - RApid Gradient Echo (MPRAGE) images being collected using GRAPPA, a parallel imaging technique based on k-space, and the following parameters: GRAPPA accel.factor PE = 2, TR = 1900 ms, TE = 2.43 ms, field of view = 256 mm, matrix size = 256 × 256, bandwidth = 180 Hz/Px, slice thickness = 1 mm, number of slices = 192, voxel size = 1 mm isotropic, and flip angle = 9°.

Functional MRI measured a blood oxygen level dependent (BOLD) signal acquired using a T2-weighted susceptibility weighted single-shot echo planar imaging (EPI) and the following parameters: TR = 1250 ms, TE = 30 ms, field of view = 256 mm, matrix size = 128 × 128, bandwidth = 1776 Hz/Px, slice thickness = 2 mm without a gap, number of slices = 56, voxel size = 2 mm isotropic, flip angle = 80°, and multi-band acceleration factor = 4. Slices were acquired interleaved from foot-to-head.

### 2.4 Data analysis

#### 2.4.1 Behavioral data analysis

Percent accuracy was evaluated for the sound and meaning task at both age 7 and age 9. For the sound task, average accuracy was calculated for “Onset” and “Rhyme” trials together. For the meaning task, average accuracy was calculated for “Low” and “High” association trials together. A child was selected for this study only if they scored greater than 50% on the perceptual and the “Rhyme” or “High” association conditions (to ensure that the child was engaged in and capable of performing the tasks). The child was selected if they had response bias, calculated as an accuracy difference between the “Rhyme” or “High” association condition (requiring a “yes” response) and the “Unrelated” condition (requiring a “no” response) of lower than 40%.

#### 2.4.2 Primary pre-registered planned analysis

##### 2.4.2.1 Preprocessing and first level modeling

The SPM12 toolbox (Statistical Parametric Mapping) (https://www.fil.ion.ucl.ac.uk/spm) was used to analyze the data. First, all functional images were realigned to their mean functional image across runs. Then, the anatomical image was segmented and warped to the appropriate pediatric tissue probability map template (Wilke et al., 2017) to obtain the transformation field. An anatomical brain mask was created by combining three segmentation products (i.e., grey, white, and cerebrospinal fluid) and then applied to its original anatomical image to produce a skull-stripped image. After that, the mean functional image and all functional images were co-registered to the skull-stripped anatomical image. All the functional images were then normalized to the pediatric template by applying the transformation field. The pediatric tissue probability map template was created using CerebroMatic (Wilke et al., 2017), a tool that makes SPM12-compatible pediatric templates by pulling a certain number of participants’ anatomical estimates generated from a publicly available dataset with user-defined age, gender, and MRI field strength. The anatomical estimates were the unified segmentation parameters estimated from 1919 participants (Wilke et al., 2017, https://www.medizin.unituebingen.de/kinder/en/research/neuroimaging/software/). The following parameters were defined to make the pediatric template: age as 7–10.5 years old with 1-month intervals, four individuals per interval with two females and two males, and magnetic field as 3 T.

To reduce movement effects on the brain signal, Art-Repair (Mazaika et al., 2009) was used to identify outlier volumes, which were defined as those with volume-to-volume head movement exceeding 1.5 mm in any direction, head movements greater than 5 mm in any direction from baseline image (first functional image), or deviations of more than 4% from the mean global signal intensity. These outlier volumes were then repaired using interpolation based on the nearest non-outlier volumes. Participants included in our final analyses had no more than 10% of the volumes and no more than six consecutive volumes repaired within each run. The movement criteria were based on those used in previous studies using the same experimental design (e.g., Wang, Yamasaki, et al., 2021; Weiss et al., 2018).

First-level statistical analysis was calculated using an event-related design with the four experimental conditions as regressors of interest. Six motion parameters estimated in the realignment step were entered in the first level modeling as regressors of no interest, and the repaired volumes were de-weighted (Mazaika et al., 2009). A high-pass temporal filter with a cutoff of 128 s and a mask threshold of 0.5 were applied. All experimental trials were included in the analysis and modeled using a canonical hemodynamic response function (HRF).

##### 2.4.2.2 Phonological and semantic specialization indexed using average beta estimates from top 100 voxels within anatomical masks for each subject [age 7 and age 9]

Anatomical ROIs were defined using the anatomical automatic labeling (AAL) atlas template using the AFNI -3dcalc command. Because the AAL atlas is based on the adult brain, the T1 structure of the AAL atlas was warped to the pediatric T1 template before selecting the anatomical ROIs. The posterior half of the left middle temporal gyrus (pMTG; y = -39), the posterior half of the left superior temporal gyrus (pSTG; y = -25), the dorsal left inferior frontal gyrus opercularis (opIFG), and the ventral left inferior frontal gyrus triangularis (trIFG) were identified as the four anatomical ROIs for further analyses. Marsbar was further used to extract the average beta values of the task-specific top 100 voxels within each anatomical ROI for each participant. Beta values are a standard measure used in the analysis of fMRI data to index the strength of task-evoked neural responses. They represent the magnitude of the BOLD signal change associated with different experimental conditions.

Average beta values extracted from the top 100 activated voxels (regardless of significance) associated with the contrast [(Low and High) – Perceptual] > [(Onset and Rhyme) – Perceptual] were used to index semantic specialization within each of the pMTG and trIFG anatomical masks for each individual. Average beta values extracted from the top 100 activated voxels associated with the contrast [(Onset and Rhyme) – Perceptual] > [ (Low and High) – Perceptual] were used to index phonological specialization within each of the pSTG and opIFG anatomical masks for each individual. For the contrast [(Onset and Rhyme) – Perceptual], the formula for setting the condition weights was (1*R1_P_O + 1*R1_P_R + 1*R2_P_O + 1*R2_P_R) - (2*R1_P_C + 2*R2_P_C), where R1 and R2 are run1 and run2, and P_O, P_R, P_C are sound task onset, rhyme and control conditions, respectively. Similarly, for the meaning task for the contrast [(Low and High) – Perceptual], the formula for setting the condition weights was (1*R1_S_H + 1*R1_S_L + 1*R2_S_H + 1*R2_S_L) - (2*R1_S_C + 2*R2_S_C), where R1 and R2 are run1 and run2, and S_H, S_L, S_C are meaning task high, low and control conditions, respectively. Semantic and phonological functional specialization was estimated at both age 7 and at age 9. The average beta values estimated at age 7 served as predictors in the hierarchical regression analysis. The average beta values estimated at age 9 served as dependent variables in the hierarchical regression analysis (Table 2). A representation map across participants showing overlap of the top 100 voxels across subjects was generated for visualizing phonological and semantic specialization within predefined anatomical masks (refer to Fig. 2A). The percentage of overlapping voxels was calculated between age 7 and age 9 for the four ROIs (refer to Fig. 2A), the percent overlapping voxels for opIFG was 95.9 %, pSTG was 85.1%, trIFG was 67.1%, and pMTG was 66.5%. Since the percentage of overlap of trIFG and pMTG was less across age 7 and age 9, supplementary analyses were conducted using consistent ROIs across time points (i.e., using the age 7 ROI definitions for both time points), based on anonymous reviewers’ comments.

**Table 2.** Hierarchical regression (HR) models to predict specialization at age 9 from fronto-temporal pathway connectivity at age 7.

	y	β_1_ x1 +	β_2_ x 2 +	β_3_ x 3 +	β_4_ x 4
	Dependent variable	Predictor variable	Control variables		
Phonological specialization					
HR1	β opIFG [age 9, phon > sem]	= Conn opIFG-pSTG [age 7, phon > P]	sound task accuracy	β opIFG [age 7, phon > sem]	β pSTG [age 7, phon > sem]
HR2	β pSTG [age 9, phon > sem]	= Conn opIFG-pSTG [age 7, phon > P]	sound task accuracy	β opIFG [age 7, phon > sem]	β pSTG [age 7, phon > sem]
Semantic specialization					
HR3	β trIFG [age 9, sem > phon]	= Conn trIFG-pMTG [age 7, sem > P]	meaning task accuracy	β trIFG [age 7, sem > phon]	β pMTG [age 7, sem > phon]
HR4	β pMTG [age 9, sem > phon]	= Conn trIFG-pMTG [age 7, sem > P]	meaning task accuracy	β trIFG [age 7, sem > phon]	β pMTG [age 7, sem > phon]

Note. The following contrast maps were used for evaluating (1) phonological specialization, phon > sem: [(Onset and Rhyme) – Perceptual] > [ (Low and High) – Perceptual], (2) semantic specialization, sem > phon: [(Low and High) – Perceptual] > [(Onset and Rhyme) – Perceptual], connectivity for (3) phonological processing, phon > P: [(Onset and Rhyme) > Perceptual] and (4) semantic processing, sem > P: [(Low and High) > Perceptual]. For the sound task average accuracy was calculated for “onset” and “rhyme” trials. For the meaning task, average accuracy was calculated for “low” and “high” association trials.

The images were visualized in MRIcroGL (https://www.nitrc.org/projects/mricrogl).

##### 2.4.2.3 Determination of task-modulated connectivity coefficients through generalized psychophysiological interactions (gPPI) [age 7]

To assess functional connectivity between fronto-temporal regions, gPPI analysis was conducted in SPM. Two gPPI models were built at the individual level with the seeds being opIFG and trIFG at age 7.

To assess functional connectivity between opIFG and pSTG during the sound task, the top 100 voxels showing maximal activation (regardless of significance) for phonological specialization estimated from the contrast [(Onset and Rhyme) – Perceptual] > [(Low and High) – Perceptual] within the anatomical mask of opIFG was used as the seed at the individual level. Next, the following regressors were entered into a general linear model (GLM) in the individual level analysis for the two runs: the time series from the seed opIFG, the 4 experimental parameter regressors (Onset, Rhyme, Unrelated, Perceptual), the PPI regressor of the interaction of seed’s activity with task condition for phonological processing (that is, opIFG x [(Onset and Rhyme) > Perceptual], and the 6 motion regressors of head movement. The task-modulated connectivity was estimated for the contrast [(Onset and Rhyme) > Perceptual] between the seed opIFG and the pSTG ROI. The pSTG ROI for each subject was defined as the top 100 voxels activated within the pSTG anatomical mask estimated for phonological specialization and was used for obtaining the connectivity coefficients for phonological processing.

To assess functional connectivity between trIFG and pMTG during the meaning task, the top 100 voxels showing maximal activation (regardless of significance) for semantic specialization estimated from the contrast [(Low and High) – Perceptual] > [(Onset and Rhyme) – Perceptual] within the trIFG anatomical mask were used as the seed at the individual level. Next, the following regressors were entered into a general linear model (GLM) in the individual level analysis for the two runs: the time series from the seed trIFG, the 4 experimental parameter regressors (High, Low, Unrelated, Perceptual), the PPI regressor of the interaction of seed’s activity with task condition for semantic processing (that is, trIFG x [(Low and High) > Perceptual], and the 6 motion regressors of head movement. The task-modulated connectivity was estimated for the contrast [(Low and High) > Perceptual] between the seed trIFG with the pMTG ROI. The pMTG ROI for each subject was defined as the top 100 voxels activated within the pMTG anatomical mask estimated for semantic specialization and was used for obtaining the connectivity coefficients for semantic processing.

##### 2.4.2.4 Hierarchical regression analyses to predict specialization at age 9 from the front-temporal pathway connectivity at age 7

Four hierarchical multiple regression analyses (HR) were performed in R (Table 2). Each HR was conducted with two blocks of predictor variables. The first block included the control variables, which include task accuracy and beta estimates for fronto-temporal regions that accounts for specialization at age 7. The task accuracy was the average accuracy for ‘Onset’ and ‘Rhyme’ trials from the sound task for hierarchical regressions 1 (HR1) and 2 (HR2) models predicting phonological specialization. For hierarchical regressions 3 (HR3) and 4 (HR4) models predicting semantic specialization, the task accuracy was the average accuracy for ‘Low’ and ‘High’ trials from the meaning task. For the second block, task-dependent functional connectivity values between fronto-temporal regions at age 7 were used as predictor variables.

The phonological specialization accounted by beta estimates in the opIFG and pSTG for the contrast [(Onset and Rhyme) – Perceptual] > [(Low and High) – Perceptual] at age 9 was entered as the dependent variable for hierarchical regressions 1 (HR1) and 2 (HR2), respectively. The semantic specialization accounted by beta estimates in the trIFG and pMTG for the contrast [(Low and High) – Perceptual] > [(Onset and Rhyme) – Perceptual] at age 9 was entered as the dependent variable for hierarchical regression 3 (HR3) and 4 (HR4), respectively (refer to Table 2). The HR3 and HR4 were repeated in supplementary analyses with specialization evaluated in trIFG and pMTG using consistent ROIs across time points (i.e., using the age 7 ROI definitions for both time points).

#### 2.4.3 Secondary pre-registered planned analysis

In primary analyses, we examined functional connectivity between the top 100 specialized voxels of IFG and the temporal lobe. In our primary pre-registered analyses, results showed that the temporal specialization was stronger and more stable (refer to Table 5). Based on this main result, we further wanted to understand if the functional connectivity of the top specialized 100 voxels in the temporal lobe with the broader language network at age 7 predicts its specialization. Thus, secondary planned analysis was further conducted to assess if network connectivity of (a) pSTG with language network at age 7 predicted phonological specialization at age 9, and (b) pMTG with language network at age 7 predicted semantic specialization at age 9. Since the focus of this study was to look at specialization of representations (not access/manipulation in frontal regions), we conducted hierarchical analyses to estimate whether the connectivity of temporal regions with the broader language network predicted later specialization within temporal regions.

##### 2.4.3.1 Defining language-sensitive masks at the individual level [age 7]

Language-sensitive masks (i.e., phonology or semantics) were defined as anatomical ROIs constrained by functional activation at the individual level. Anatomical ROIs were identified using the anatomical automatic labeling (AAL) atlas template using the AFNI -3dcalc command. Because the AAL atlas is based on the adult brain, the T1 structure of the AAL atlas was warped to the pediatric T1 template before selecting the anatomical ROIs. Three anatomical ROIs were defined as follows: The frontal anatomical ROI comprised the dorsal left inferior frontal gyrus opercularis (opIFG), the ventral left inferior frontal gyrus triangularis (trIFG), and the left inferior frontal gyrus orbitalis (orbIFG); The temporal anatomical ROI comprised left temporal regions, including the anterior half of the middle temporal gyrus (aMTG; y = -39), the anterior half of the superior temporal gyrus (aSTG; y = -25); the parietal anatomical ROI comprised of left parietal regions including the supramarginal gyrus (SMG), angular gyrus (AG), and inferior parietal lobule (IPL). The phonological-sensitive masks were defined at the individual level as the top 1000 activated voxels for the contrast (Onset + Rhyme > Perceptual) within each of the frontal, temporal, and parietal anatomical ROIs. The semantic-sensitive masks were defined at the individual level as the top 1000 activated voxels for the contrast (Low+ High > Perceptual) within each of the frontal, temporal, and parietal anatomical ROIs.

##### 2.4.3.2 Determination of task-modulated connectivity coefficients through generalized psychophysiological interactions (gPPI) [age 7]

Two gPPI models were built at the individual level to determine task-related connectivity with the seeds being pSTG and pMTG at age 7.

To assess functional connectivity between pSTG and language mask during the sound task, the top 100 voxels showing maximal activation (regardless of significance) for phonological specialization estimated from the contrast [(Onset and Rhyme) – Perceptual] > [(Low and High) – Perceptual] within the anatomical mask of pSTG were used as the seed at the individual level. Task-modulated connectivity was computed between the seed pSTG and (1) frontal (2) temporal and (3) parietal, phonological-sensitive ROIs for phonological processing. The following regressors were entered into a general linear model (GLM) in the individual level analysis for the two runs: the time series from the seed pSTG, the 4 experimental parameter regressors (Onset, Rhyme, Unrelated, Perceptual), the PPI regressor of the interaction of seed activity with task condition for phonological processing (that is, pSTG x [(Onset and Rhyme) > Perceptual], and the 6 motion regressors of head movement. The task-modulated connectivity was estimated for the contrast [(Onset and Rhyme) > Perceptual] between the seed pSTG and the (1) frontal, (2) temporal, and (3) parietal, phonological-sensitive ROIs. The (1) frontal, (2) temporal, and (3) parietal, phonological-sensitive ROIs for each participant were defined as the top connected 100 voxels within the participant’s phonological-sensitive top 1000 activated voxel masks and were used to obtain functional connectivity coefficients for phonological processing. A visualization map was plotted for the top 100 voxels connected with pSTG within the phonological sensitive masks overlapped across subjects (refer to Fig. 4A). The images were visualized in MRIcroGL (https://www.nitrc.org/projects/mricrogl).

To assess functional connectivity between pMTG and language mask during the meaning task, the top 100 voxels showing maximal activation (regardless of significance) for semantic specialization estimated from the contrast [(Low and High) – Perceptual] > [(Onset and Rhyme) – Perceptual] within the anatomical mask of pMTG were used as the seed at the individual level. Task-modulated connectivity was computed between the seed pMTG and (1) frontal, (2) temporal, and (3) parietal, semantic-sensitive ROIs for semantic processing. The following regressors were entered into a general linear model (GLM) in the individual level analysis for the two runs: the time series from the seed pMTG, the 4 experimental parameter regressors (High, Low, Unrelated, Perceptual), the PPI regressor of the interaction of seed activity with task condition for semantic processing (that is, pMTG x [(Low and High) – Perceptual], and the 6 motion regressors of head movement. The task-modulated connectivity was estimated for the contrast [(Low and High) – Perceptual] between the seed pMTG and the (1) frontal, (2) temporal, and (3) parietal, semantic-sensitive ROIs. The (1) frontal, (2) temporal, and (3) parietal, semantic-sensitive ROIs for each participant were defined as the top connected 100 voxels within the participant’s semantic-sensitive top 1000 activated voxel masks and were used to obtain functional connectivity coefficients for semantic processing. A visualization map was plotted for the top 100 voxels connected with pMTG within the semantic-sensitive masks overlapped across subjects (refer to Fig. 4B).

##### 2.4.3.3 Hierarchical regression analyses to predict specialization at age 9 from network connectivity of temporal regions at age 7

Six hierarchical regressions (HR) analyses were computed to estimate whether the connectivity of temporal regions with the language network predicted later specialization of the temporal regions (Table 3). The first block included the control variables such as the task accuracy and beta estimate for temporal specialization at age 7. The task accuracy was average accuracy for ‘Onset’ and ‘Rhyme’ trials from the sound task for hierarchical regressions 1 (HR1), 2 (HR2), and 3 (HR3) models predicting phonological specialization. For hierarchical regressions 4 (HR4), 5 (HR5), and 6 (HR6) models predicting semantic specialization, the task accuracy was average accuracy for ‘Low’ and ‘High’ related trials from the meaning task (refer to Table 3).

**Table 3.** Hierarchical regression (HR) models to predict specialization at age 9 from network connectivity of temporal regions at age 7.

Y Dependent		β_1_ x1 Predictor variable	+ β_2_ x 2 + β_3_ x 3 + β_4_ x 4 + β_5_ x 5 Control variables			
Phonological specialization						
HR1	β pSTG [age 9, phon > sem]	= Conn pSTG-frontal [age 7, phon > P]	Conn pSTG-temporal [age 7, phon > P]	Conn pSTG-parietal [age 7, phon > P]	Sound task accuracy	β pSTG [age 7, phon > sem]
HR2	β pSTG [age 9, phon > sem]	= Conn pSTG-temporal [age 7, phon > P]	Conn pSTG-frontal [age 7, phon > P]	Conn pSTG-parietal [age 7, phon > P]	Sound task accuracy	β pSTG [age 7, phon > sem]
HR3	β pSTG [age 9, phon > sem]	= Conn pSTG-parietal [age 7, phon > P]	Conn pSTG-frontal [age 7, phon > P]	Conn pSTG-temporal [age 7, phon > P]	Sound task accuracy	β pSTG [age 7, phon > sem]
Semantic specialization						
HR4	β pMTG [age 9, sem > phon]	= Conn pMTG-frontal [age 7, sem > P]	Conn pMTG-temporal [age 7, sem > P]	Conn pMTG-parietal [age 7, sem > P]	Meaning task accuracy	β pMTG [age 7, sem > phon]
HR5	β pMTG [age 9, sem > phon]	= Conn pMTG-temporal [age 7, sem > P]	Conn pMTG-frontal [age 7, sem > P]	Conn pMTG-parietal [age 7, sem > P]	Meaning task accuracy	β pMTG [age 7, sem > phon]
HR6	β pMTG [age 9, sem > phon]	= Conn pMTG-parietal [age 7, sem > P]	Conn pMTG-frontal [age 7, sem > P]	Conn pMTG-temporal [age 7, sem > P]	Meaning task accuracy	β pMTG [age 7, sem > phon]

Note. The following contrast maps were used for evaluating (1) phonological specialization, phon > sem: [(Onset and Rhyme) – Perceptual] > [ (Low and High) – Perceptual], (2) semantic specialization, sem > phon: [(Low and High) – Perceptual] > [(Onset and Rhyme) – Perceptual], connectivity for (3) phonological processing, phon > P: [(Onset and Rhyme) > Perceptual] and (4) semantic processing, sem > P: [(Low and High) > Perceptual]. For the sound task average accuracy was calculated for “onset” and “rhyme” trials. For the meaning task, average accuracy was calculated for “low” and “high” association trials.

The task-modulated connectivity estimates between the seed pSTG and the (1) frontal (2) temporal and (3) parietal, phonological-sensitive ROIs during phonological processing were used as predictor variables in HR1, HR2, and HR3, respectively. The task-modulated connectivity estimates were computed between the seed pMTG and the (1) frontal, (2) temporal, and (3) parietal, semantic-sensitive ROIs during semantic processing were used as predictor variables in HR4, HR5, and HR6, respectively

The connectivity estimates with other non-interest ROIs were used as control variables to estimate the variance explained by the ‘connection of interest’ in the HR models (for example, for HR1, the connectivity estimate of the pSTG-frontal was the predictor variable and the connectivity estimates for pSTG-temporal and pSTG-parietal were the control variables, refer to Table 3 for all other models). The phonological specialization accounted by beta estimates for pSTG by the contrast [(Onset and Rhyme) – Perceptual] > [(Low and High) – Perceptual] at age 9 was entered as the dependent variable for hierarchical regressions 1 (HR1), 2 (HR2), and 3 (HR3). The semantic specialization accounted by beta estimates for pMTG for the contrast [(Low and High) – Perceptual] > [(Onset and Rhyme) – Perceptual] at age 9 was entered as the dependent variable for hierarchical regression 4 (HR4), 5 (HR5) and 6 (HR6).

## 3 Results

### 3.1 Behavioral accuracy on in-scanner tasks

**Fig. 1.**
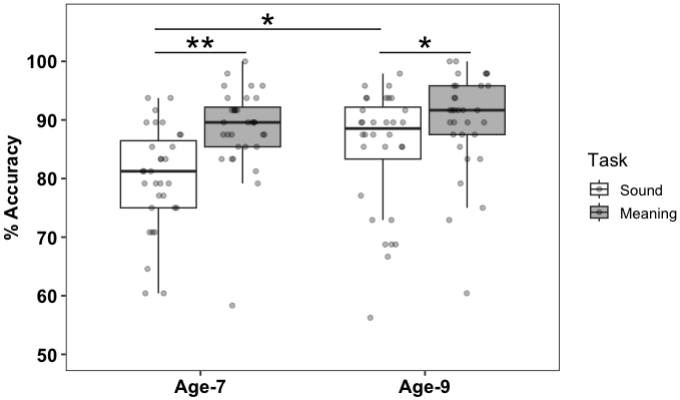
Behavioral Accuracy on In-Scanner Tasks. The box plots represent the percent accuracy in sound and meaning task for 32 participants at both age 7 and age 9. The box plots used in all figures represent the median values (dark black line at the center of each box). The lower and upper lines of each box correspond to the first and third quartiles (the 25th and 75th percentiles). The upper whisker extends from the box to the largest value no further than 1.5 times inter-quartile range (IQR, or distance between the first and third quartiles). The lower whisker extends from the box to the smallest value at most 1.5 times IQR. Individual data points are jittered on top of each box. **p < 0.001, *p < 0.05 p-value adjusted for multiple comparisons, Bonferroni correction for two comparisons.

Figure 1 shows the box plots representing the percent accuracy in sound and meaning tasks for 32 participants at both age 7 and age 9. Children performed better at the meaning task as compared to the sound task at both age 7 (t (31) = -5.46, p < 0.001, adjusted p-value reported after Bonferroni correction for two comparisons) and age 9 (t (31) = -2.49, p = 0.04, adjusted p-value reported after Bonferroni correction for two comparisons). At age 7, the average accuracy for the meaning task was 88.61% ± 7.37%, and the average accuracy for the sound task was 79.10 % ± 10.19%. At age 9, the average accuracy for the meaning task was 89.78% ± 8.55%, and the average accuracy for the sound task was 85.09% ± 10.37%. The developmental change in task accuracy across age 7 and age 9 was significant for the sound task (t (31) = -3.10, p = 0.004, adjusted p-value reported after Bonferroni correction for two comparisons) but not for the meaning task (t (31) = -0.61, p = 1.00, adjusted p-value reported after Bonferroni correction for two comparisons).

### 3.2 Primary pre-registered analysis

#### 3.2.1 Phonological and semantic specialization at age 7 and age 9

We evaluated beta estimates for phonological specialization and semantic specialization (post-hoc analysis based on anonymous reviewers’ comments). The results are reported in Supplementary Table 1.

**Fig. 2.**
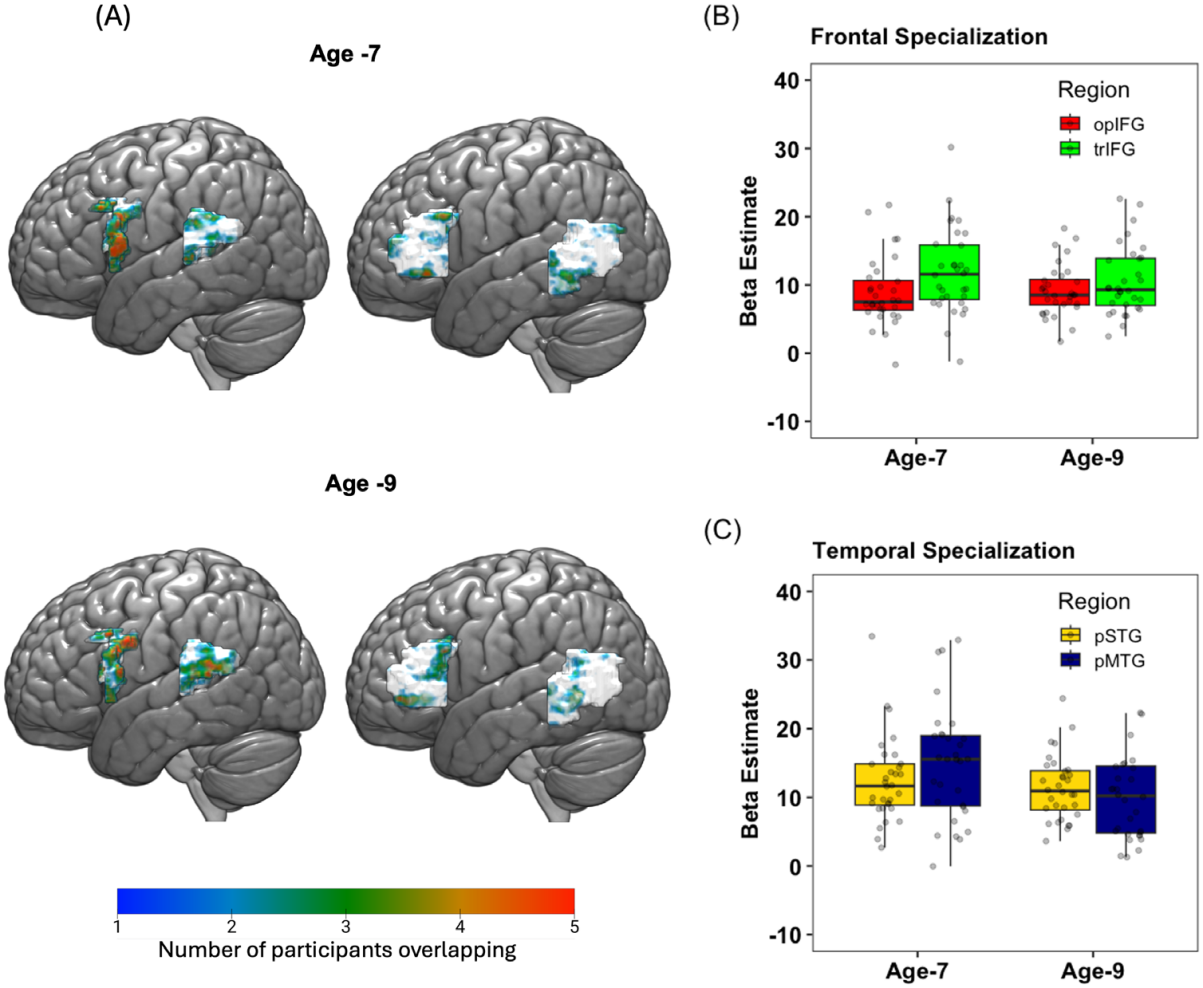
Phonological and semantic specialization at age 7 and age 9. The brain maps represent the overlap of top 100 voxels within the anatomical masks for phonological specialization [(Onset and Rhyme) – Perceptual] > [ (Low and High) – Perceptual] in opIFG and pSTG and semantic specialization [(Low and High) – Perceptual] > [(Onset and Rhyme) – Perceptual] in trIFG and pMTG at both age 7 and age 9. The color gradient shows the number of participants with overlapping voxels (A). The barplots in (B) represent the beta estimate values for phonological specialization in opIFG (red) and for semantic specialization in trIFG (green) at both age 7 and age 9. The barplots in (C) represent the beta estimate values for phonological specialization in pSTG (yellow) and for semantic specialization in pMTG (blue) at both age 7 and age 9.

At age 7, higher activity was observed in trIFG for semantic specialization (average beta estimate 11.918 ± 6.241) as compared to opIFG for phonological specialization (average beta estimate 8.883 ± 4.981). Moreover, at age 7, higher activity was observed in pMTG for semantic specialization (average beta estimate 15.903 ± 9.883) than pSTG for phonological specialization (average beta estimate 12.442 ± 6.167) (Fig. 2B). Although higher activity was observed for semantic specialization as compared to phonological specialization at age 7, the results of paired t-test revealed that the difference was not significant. At age 9, no significant difference was observed in fronto-temporal phonological and semantic specialization (refer to Fig. 2 and Table 4).

**Table 4.** Average beta estimate (n = 32) for phonological specialization within opIFG and pSTG and for semantic specialization within trIFG and pMTG at both age 7 and age 9.

Age-7			Age-9		
Phonological specialization	Semantics pecialization	Paired t-test (p-value)	Phonological specialization	Semantics pecialization	Paired t-test (p-value)
OpIFG	trIFG		opIFG	trIFG	
8.883 ± 4.981	11.918 ± 6.241	t (31) = -1.77, p = 0.17	9.157 ± 3.774	10.580 ± 5.061	t (31) = -1.10, p = 0.56
Pstg	pMTG		pSTG	pMTG	
12.442 ± 6.167	15.903 ± 9.883	t (31) = 1.50, p = 0.29	11.376 ± 4.691	11.115 ± 8.256	t (31) = -0.15, p = 1.00

Note. The results of paired t-tests comparisons comparing phonological and semantic specialization are reported with p- value adjusted for multiple comparisons, Bonferroni correction for two comparisons.

#### 3.2.2 Task-modulated connectivity at age 7

**Fig. 3.**
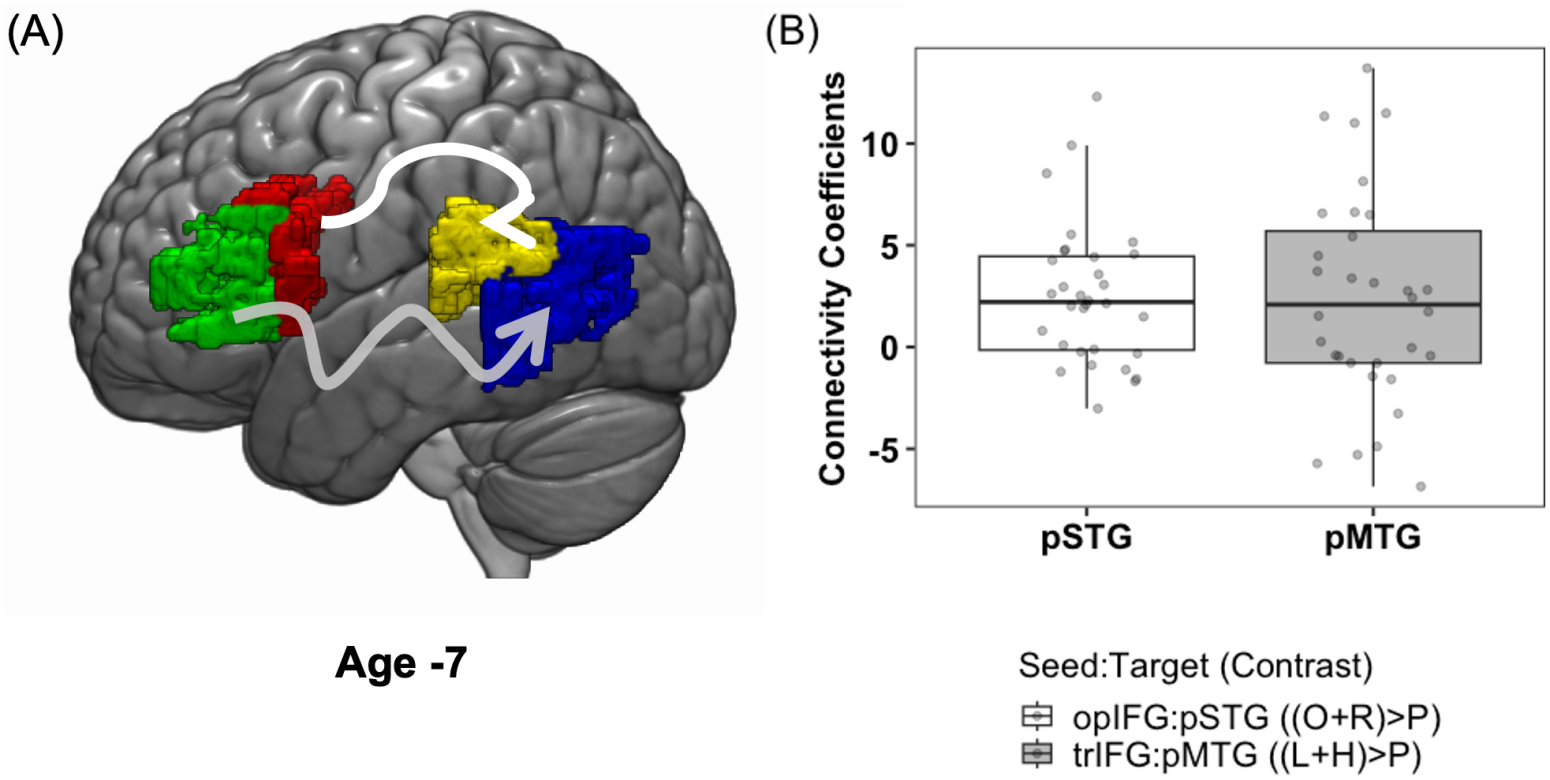
Task-modulated pathway connectivity at age 7. The brain map shows a schematic of the two gPPI models built at individual level with seed region of interests opIFG (red) and trIFG (green) for obtaining connectivity coefficients for phonological and semantic processing, represented with white and grey arrows respectively (A). The barplots in (B) represent the task-modulated connectivity coefficients within pSTG for phonological processing [(Onset and Rhyme) – Perceptual] (white) and within pMTG for semantic processing [(Low and High) – Perceptual] (grey).

The paired t-test results indicated no significant difference between the connectivity estimates computed between opIFG seed and pSTG for phonological processing (average connectivity estimate 2.55 ± 3.42) and the connectivity estimates computed between trIFG seed and pMTG for semantic processing (average connectivity estimate 2.35 ± 5.23), t (31) = 0.18, p = 0.86 (refer to Fig. 3).

#### 3.2.3 Predicting specialization at age 9 from pathway connectivity at age 7

Four hierarchical regression analyses were conducted to predict fronto-temporal phonological and semantic specialization at age 9 from fronto-temporal connectivity at age 7 (controlling for task accuracy and specialization at age 7). Specifically, we evaluated the functional connectivity of the frontal lobe to the temporal lobe at age 7 and addressed (a) if the connectivity between dIFG and pSTG at age 7 predicted phonological specialization at age 9 in opIFG (HR1) and pSTG (HR2) and (b) if the connectivity between vIFG and pMTG at age 7 predicted semantic specialization at age 9 in trIFG (HR3) and pMTG (HR4). Results show that overall opIFG-pSTG connectivity was a significant predictor for phonological specialization in pSTG at age 9 (β = -0.633, p = 0.02). Moreover, phonological specialization in opIFG at age 7 predicted specialization in pSTG at age 9 (β = -0.42, p = 0.04), whereas semantic specialization in pMTG at age 7 significantly predicted specialization in trIFG (β = 0.40, p < 0.01) and pMTG at age 9 (β = 0.71, p < 0.01) (refer to Table 5). However, trIFG-pMTG connectivity was not a significant predictor for semantic specialization in pMTG at age 9.

**Table 5.** Hierarchical regression (HR) analyses results to predict specialization at age 9 from fronto-temporal pathway connectivity at age 7.

1. Dependent measure (phonological specialization)					
		HR1 (β opIFG Age 9)		HR2 (β pSTG Age 9)	
	Predictors (Age 7)	β	p	β	p
Model 1	Sound task accuracy	3.243	0.643	6.971	0.416
	β opIFG	0.134	0.422	-0.255	0.215
	β pSTG	-0.101	0.454	0.0931	0.570
R^2^ (ΔR^2^)		0.037 (-0.066)		0.07 (-0.029)	
Model 2	Sound task accuracy	2.447	0.740	1.564	0.848
	β opIFG	0.110	0.543	**-0.422**	0.041*
	β pSTG	-0.091	0.509	0.156	0.310
	Conn opIFG-pSTG	-0.093	0.690	**-0.633**	0.020*
R^2^ (ΔR^2^)		0.043 (-0.098)		0.243 (0.13)	

2. Dependent measure (Semantic specialization)					
		HR3 (β trIFG Age 9)		HR4 (β pMTG Age 9)	
	Predictors (Age 7)	β	p	β	p
Model 1	Meaning task accuracy	15.906	0.193	4.074	0.830
	β trIFG	-0.418	0.062	-0.582	0.099
	β pMTG	**0.391**	0.009**	**0.665**	0.005**
R^2^ (ΔR^2^)		0.228 (0.145)	0.276 (0.199)
Model 2	Meaning task accuracy	14.802	0.246	-1.2471	0.948
	β trIFG	-0.435	0.062	-0.6591	0.067
	β pMTG	**0.401**	0.009**	**0.7138**	0.004**
	Conn trIFG-pMTG	-0.063	0.720	-0.305	0.269
R^2^ (ΔR2)		0.232 (0.118)		0.309 (0.206)	

Note. The first table panel has the results of hierarchical regression analysis to predict phonological specialization in opIFG (HR1) and pSTG (HR2) at age 9 from fronto-temporal connectivity at age 7 (model 2) while controlling for task accuracy and early phonological specialization in opIFG and pSTG at age 7 (model 1). The second table panel has the results of hierarchical regression analysis to predict semantic specialization in trIFG (HR3) and pMTG (HR4) at age 9 from fronto-temporal connectivity at age 7 (model 2) while controlling for task accuracy and early semantic specialization in trIFG and pMTG at age 7 (model 1). Statistically significant results are shown in bold (*p < .05, **p < .01).

Based on anonymous reviewers’ comments, to assess the null findings for connectivity predicting specialization in semantic processing regions, supplementary analyses were conducted using consistent ROIs across time points (i.e., using the age 7 ROI definitions for both time points) (Table 6). Using age 7 ROIs, connectivity betas were extracted for age 9 for semantic specialization in trIFG and pMTG; thereafter, HR3 and HR4 analyses were reconducted with these new values of specialization calculated at age 9. Results show that overall trIFG-pMTG connectivity was a significant predictor for semantic specialization in pMTG at age 9 (β = -0.42, p = 0.035) (Table 6).

**Table 6.** Hierarchical regression (HR) analyses results to predict semantic specialization at age 9 from fronto-temporal pathway connectivity at age 7 using consistent ROIs across age 7 and age 9.

1. Dependent measure (semantic specialization)					
		HR3 (β trIFG Age 9)		HR4 (β pMTG Age 9)	
	Predictors (Age 7)	β	p	β	p
Model 1	Meaning task accuracy	8.573	0.516	-25.393	0.081
	β trIFG	-0.377	0.121	0.088	0.731
	β pMTG	0.236	0.132	0.033	0.842
R^2^ (ΔR^2^)		0.093 (-0.004)		0.144 (0.052)	
Model 2	Meaning task accuracy	9.749	0.481	**-32.726**	0.023*
	β trIFG	-0.340	0.152	-0.019	0.938
	β pMTG	0.226	0.164	0.101	0.526
	Conn trIFG-pMTG	0.067	0.727	**-0.420**	0.035*
R^2^ (ΔR2)		0.097 (-0.037)		0.276 (0.168)	

Note. Using age 7 ROIs, connectivity betas were extracted for age 9 for semantic specialization in trIFG and pMTG. Table 6 has the results of hierarchical regression analysis conducted to predict semantic specialization in trIFG (HR3) and pMTG (HR4) at age 9 (evaluated using age 7 ROIs), from fronto-temporal connectivity at age 7 (model 2) while controlling for task accuracy and early semantic specialization in trIFG and pMTG at age 7 (model 1). Statistically significant results are shown in bold (*p < .05).

### 3.3 Secondary pre-registered analysis

#### 3.3.1 Task-modulated connectivity at age 7 within language mask

**Fig. 4.**
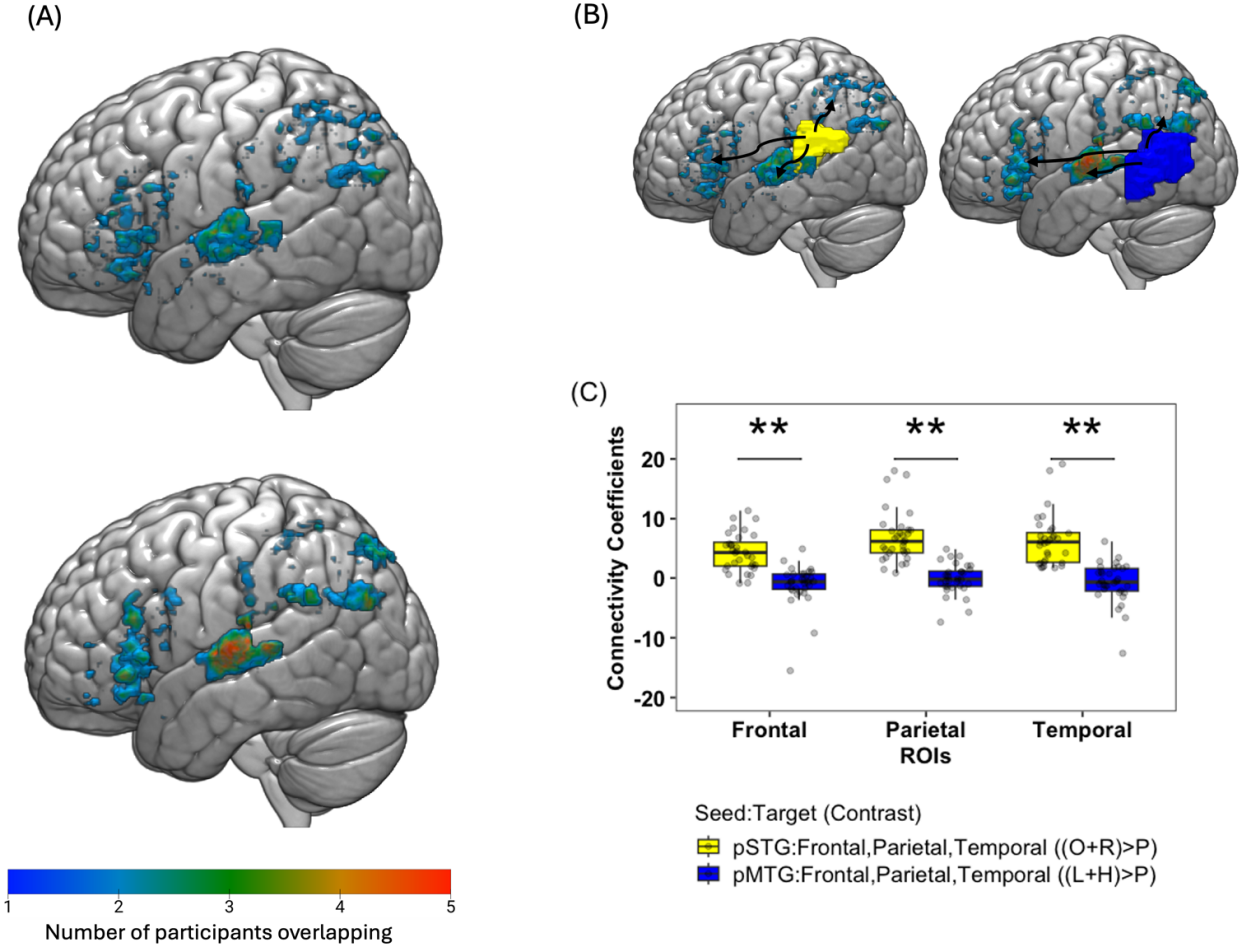
Task-modulated network connectivity at age 7 within language mask. The brain maps represent the overlap of top 100 connected voxels within frontal, temporal and parietal language masks with pSTG (top panel) and pMTG (bottom panel) in (A). The color gradient shows the number of participants with overlapping voxels (A). The brain map in (B) shows a schematic of the two gPPI models built at individual level with seed region of interests pSTG (yellow) and pMTG (blue) with the language mask for obtaining connectivity coefficients for phonological processing [(Onset and Rhyme) – Perceptual] and semantic processing [(Low and High) – Perceptual]. The barplots represent the task-modulated connectivity coefficients for frontal, parietal, and temporal ROIs with pSTG as seed (yellow) and pMTG as seed (blue) at age 7 (C). **p < 0.001, adjusted p-value adjusted for multiple comparisons, Bonferroni correction for three comparisons.

The paired t-test results indicate significant differences between the connectivity estimates of pSTG seed and pMTG seed with frontal, temporal, and parietal ROIs. Higher connectivity was observed for pSTG with frontal (average connectivity estimate 4.34 ± 3.19), parietal (6.87 ± 4.28) and temporal (6.31 ± 4.30) phonological sensitive ROIs as compared to connectivity of pMTG with frontal (-1.13 ± 3.53), parietal (-0.12 ± 2.59), and temporal semantic sensitive ROIs (-0.74 ± 3.41), (t (31) = -6.44, p < 0.001; t (31) = -7.42, p < 0.001; t (31) = -8.02, p < 0.001, adjusted p-value reported after Bonferroni correction for three comparisons) (refer to Fig. 4).

#### 3.3.2 Predicting specialization at age 9 from network connectivity at age 7

Six hierarchical regression analyses were conducted to predict temporal specialization at age 9 from network connectivity of temporal regions at age 7 (controlling for connectivity with other regions within the language mask, task accuracy and specialization at age 7). Results indicate that overall connectivity is not a significant predictor of specialization. However, we find that semantic specialization in pMTG at age 7 significantly predicts specialization in pMTG at age 9 in all the hierarchical regression models (refer to Table 7).

**Table 7.** Hierarchical regression (HR) analyses to predict specialization at age 9 from network connectivity of temporal regions at age 7.

1. Dependent measure (phonological specialization)									
	HR1 (β pSTG age 9)			HR2 (β pSTG age 9)			HR3 (β pSTG age 9)		
	Predictors (Age 7)	β	p	Predictors (Age 7)	β	p	Predictors (Age 7)	β	p
Model 1	Sound task acc	8.391	0.392	Sound task acc	10.360	0.274	Sound task acc	4.915	0.582
	β pSTG	-0.047	0.757	β pSTG	-0.012	0.933	β pSTG	-0.021	0.889
	Conn pSTG - temporal	-0.088	0.708	Conn pSTG - frontal	0.518	0.166	Conn pSTG - frontal	0.225	0.454
	Conn pSTG - parietal	-0.133	0.602	Conn pSTG - parietal	-0.436	0.144	Conn pSTG - temporal	-0.180	0.419
R^2^(ΔR^2^)	0.042 (-0.100)			0.104 (-0.029)			0.052 (-0.088)		
Model 2	Sound task acc	9.868	0.310	Sound task acc	9.869	0.310	Sound task acc	9.869	0.310
	β pSTG	-0.023	0.878	β pSTG	-0.023	0.878	β pSTG	-0.023	0.878
	Conn pSTG - temporal	-0.081	0.729	Conn pSTG - frontal	0.514	0.176	Conn pSTG with frontal	0.514	0.176
	Conn pSTG - parietal	-0.399	0.213	Conn pSTG - parietal	-0.399	0.213	Conn pSTG temporal	-0.081	0.729
	Conn pSTG - frontal	0.514	0.176	Conn pSTG - temporal	-0.081	0.729	Conn pSTG parietal	-0.399	0.213
R^2^(ΔR^2^)	0.108 (-0.064)			0.108 (-0.063)			0.108 (-0.063)		

2. Dependent measure (semantic specialization)									
	HR4 (β pMTG Age 9)			HR5 (β pMTG Age 9)			HR6 (β pMTG Age 9)		
	Predictors (Age 7)	β	p	Predictors (Age 7)	β	p	Predictors (Age 7)	β	p
Model 1	Meaning task acc	-4.750	0.807	Meaning task acc	-4.324	0.827	Meaning task acc	-1.827	0.927
	β pMTG	**0.387**	0.014*	β pMTG	**0.365**	0.017*	β pMTG	**0.403**	0.012*
	Conn pMTG - temporal	-0.257	0.563	Conn pMTG - frontal	0.064	0.873	Conn pMTG - frontal	0.131	0.756
	Conn pMTG - parietal	-0.551	0.341	Conn pMTG - parietal	-0.672	0.224	Conn pMTG - temporal	-0.440	0.323
R^2^(ΔR^2^)	0.253 (0.142)			0.244 (0.132)			0.229 (0.116)		
Model 2	Meaning task acc	-3.899	0.845	Meaning task acc	-3.899	0.845	Meaning task acc	-3.899	0.845
	β pMTG	**0.388**	0.015*	β pMTG	**0.388**	0.015*	β pMTG	**0.388**	0.015*
	Conn pMTG - temporal	-0.296	0.528	Conn pMTG - frontal	0.134	0.751	Conn pMTG - frontal	0.134	0.751
	Conn pMTG - parietal	-0.552	0.348	Conn pMTG - parietal	-0.552	0.348	Conn pMTG - temporal	-0.296	0.528
	Conn pMTG - frontal	0.134	0.751	Conn pMTG - temporal	-0.296	0.528	Conn pMTG - parietal	-0.552	0.348
R^2^(ΔR^2^)	0.256 (0.113)			0.256 (0.113)			0.256 (0.113)		

Note. The first table panel has the results of three hierarchical regression analysis to predict phonological specialization in pSTG at age 9 from pSTG connectivity with frontal (HR1), temporal (HR2), and parietal (HR3) phonological-sensitive ROIs at age 7 (model 2) while controlling for task accuracy, early phonological specialization in pSTG, and connectivity estimates with other non-interest ROIs at age 7 (model 1). The second table panel has the results of three hierarchical regression analysis to predict semantic specialization in pMTG at age 9 from pMTG connectivity with frontal (HR1), temporal (HR2), and parietal (HR3) semantic-sensitive ROIs at age 7 (model 2) while controlling for task accuracy, early semantic specialization in pMTG, and connectivity estimates with other non-interest ROIs at age 7 (model 1). Statistically significant results are shown in bold (*p < .05).

## 4 Discussion

This study evaluates a core hypothesis of the interactive specialization model (ISM) that early brain connectivity in young children sets the stage for later functional specialization (Johnson, 2011). In primary pre-registered analyses, we used hierarchical regression models to determine if early fronto-temporal connectivity in the dorsal and the ventral language pathway at age 7 predicts later specialization at age 9 during a sound and a meaning task. This preregistered analysis showed that earlier specialization in opIFG and opIFG-pSTG connectivity within the dorsal pathway were significant predictors of later phonological specialization. This preregistered analysis also showed in the ventral pathway, specialization but not connectivity at age 7 was a predictor of semantic specialization at age 9. However, a supplementary analysis using consistent ROIs across time points (age 7–defined ROIs applied to both time points) did reveal that earlier trIFG–pMTG connectivity predicted later semantic specialization in pMTG. In secondary pre-registered analyses, we further evaluated whether connectivity between the temporal cortex and an extended language network at age 7 predicted specialization at age 9. In these network analyses, the connectivity of temporal cortex with the broader language network at age 7 did not predict phonological or semantic specialization at age 9. As with pathway analyses, the network analyses showed that age 7 semantic specialization significantly predicted age 9 semantic specialization. As opposed to the pathway analyses, the network analyses did not show a predictive effect of earlier on later phonological specialization. In summary, our results support a core hypothesis of ISM (Johnson, 2011) with the evidence that opIFG-pSTG connectivity appeared as a significant predictor for subsequent phonological specialization, and trIFG–pMTG connectivity appeared as a significant predictor for subsequent semantic specialization.

### 4.1 Earlier connectivity in the dorsal pathway predicted later temporal specialization during phonological processing

The findings from the hierarchical regression analyses demonstrate a relationship between fronto-temporal connectivity in the dorsal pathway at age 7 and phonological specialization in the pSTG at age 9. These variations in earlier connectivity are significant predictors of later phonological specialization, even when controlling for earlier task accuracy and specialization. The negative beta coefficient suggests an inverse relationship whereby children who demonstrate higher levels of pathway connectivity at age 7 exhibit less specialized phonological systems by age 9. Prior research has implied the role of the inferior frontal gyrus (IFG) in modulating the sensitivity of the posterior cortex for processing various types of inputs (Bitan et al., 2005, 2006), which may explain why fronto-temporal connectivity predicts specialization in pSTG. Phonological processing was measured by a sound task that required participants to determine whether the onset or rhyme matched between a pair of auditorily presented words. The sound task requires segmentation of the components of words and maintaining them over time to make a comparison. Participants performed the sound task with lower accuracy compared to the meaning task at both ages 7 and age 9, and only the sound task showed a developmental increase in accuracy. The sound task’s meta-linguistic demands likely contribute to the observed lower accuracy scores and more pronounced developmental increase. Overall, the finding for the sound task is consistent with a core assumption of the ISM, which posits a dynamic interaction between connectivity and specialization over time (Johnson, 2011).

Although the pathway analysis showed that earlier connectivity predicted later specialization for the sound task, the network analysis failed to show this effect. However, it is important to note that the frontal connectivity in the network analysis exhibited the largest beta coefficients. The lack of predictive power in the network analyses may be attributed to the relatively widespread nature of the connected voxels. The pathway analysis examined connectivity between pSTG and the opercularis region of IFG. In contrast, the network analyses examined connectivity between the pSTG and both the opercularis and triangularis regions of IFG. Examination of the connected voxels used in the network analyses shows both dorsal and ventral regions of the IFG, which suggests the engagement of multiple underlying mechanisms. The inclusion of these multiple underlying mechanisms may have masked the effect of earlier fronto-temporal connectivity on later temporal specialization in the sound task.

### 4.2 Earlier specialization predicted later specialization more for semantic compared to phonological processing

Specialization in the pMTG at age 7 significantly predicted frontal and temporal specialization at age 9 in the pathway analyses and temporal specialization in the network analyses. These effects were robust when compared to the localized effect of IFG specialization at age 7, predicting pSTG specialization for the sound task only in the pathway analyses. While we are not aware of previous studies that have demonstrated a predictive relation of earlier to later specialization, longitudinal research has indicated that early specialization of temporal regions for semantic processing, particularly in the pMTG, is predictive of later language and reading outcomes in children (Lee et al., 2016) and early specialization of temporal regions for phonological processing, particularly in the pSTG is predictive of reading growth (Yamasaki et al., 2021). The observed stability in semantic specialization across ages 7 and 9 in the temporal lobe may be attributed to the better performance on the meaning task. There were no developmental changes on the meaning task, and accuracy was higher on the meaning task compared to the sound task.

### 4.3 Earlier connectivity in the ventral pathway predicted later temporal specialization during semantic processing

In our preregistered primary ventral pathway analysis, trIFG–pMTG connectivity at age 7 did not predict later semantic specialization. However, the supplementary analysis using consistent ROIs across time points (Table 6) showed that earlier trIFG–pMTG connectivity did predict later pMTG specialization. This convergence with the dorsal pathway finding strengthens support for ISM by indicating that, when ROI definitions were consistent over time, early ventral pathway connectivity also forecasted later semantic specialization. As with the dorsal finding, the negative beta suggests that greater early pathway connectivity relates to less specialization in pMTG at later age. This may reflect a developmental trajectory in which children with higher early coupling rely on broader, more integrated processing that becomes more selective at different rates across individuals.

### 4.4 Age and domain differences in interactive specialization

The ISM proposes that functional specialization emerges gradually over the course of development. As noted above, we showed relatively modest evidence for earlier connectivity predicting later specialization from 7- to 9-year-olds. Connectivity-to-specialization effects were detected in the pathway analyses for phonology (primary) and for semantics under a consistent-ROI supplementary analysis, but not in the network analyses. Prior research addressing the ISM has produced inconclusive evidence in support of it. Some longitudinal studies examining both connectivity and specialization have not directly tested for a relation between them (Pleisch et al., 2019). For those that have examined this relation, some have failed to find one, such as Battista (2018) who examined number processing in the parietal lobe. Others have shown that earlier connectivity predicts later specialization for face versus object processing in occipitotemporal cortex (O’Rawe et al., 2019) and for word versus object processing in the visual word form area (Saygin et al., 2016). The divergent findings across studies may reflect the variable developmental trajectories of interactive specialization across different neurocognitive domains. The inconsistency could also be due to varying approaches for assessing functional connectivity. Both studies (O’Rawe et al., 2019; Saygin et al., 2016) that observed a relationship between connectivity and regional specialization characterized connectivity patterns of the targeted regions by widespread connections to multiple brain regions. In contrast, we found that earlier connectivity predicted later phonological and semantic specialization for the pathway but not the network analyses, the latter of which included widespread language regions. Future work should investigate whether earlier connectivity predicts later specialization in different age groups in different cognitive domains using a variety of analytical approaches.

### 4.5 Conclusion

We found converging evidence for a core ISM prediction: earlier functional connectivity forecasted later specialization in childhood language networks. In preregistered pathway analyses, dorsal opIFG–pSTG connectivity predicted later phonological specialization in pSTG. Supplementary analyses with consistent ROIs further showed that ventral trIFG–pMTG connectivity predicted later semantic specialization in pMTG. Network-level connectivity to broader language regions did not predict later specialization, underscoring the importance of pathway specificity. Robust stability of semantic specialization from ages 7 to 9 likely reflects the easier task demands of the meaning task. Future studies should test younger cohorts, manipulate task difficulty (particularly for semantics), and integrate structural connectivity to assess whether white-matter organization similarly constrains the emergence of functional specialization. We are currently in the process of examining the relation of dorsal and ventral white matter pathways to phonological and semantic skills (https://osf.io/dm78z). In the future, we also plan to investigate whether these structural pathways are related to fronto-temporal phonological and semantic specialization (https://osf.io/cfgr2).

## Supplementary Material

Supplementary Material

## Data Availability

All data were pulled from a published data set on OpenNeuro.org (see detailed description in Wang et al. (2022); https://openneuro.org/datasets/ds003604). All analyses were preregistered on Open Science Framework (OSF) (see https://osf.io/6ecyr). The preregistration was submitted after checking the inclusionary criteria to determine the sample size for this longitudinal study. The specific subjects and runs used in the current study as well as the code used to analyze the data were shared on GitHub (https://github.com/avantika9/ELP_Specialization_Connectivity_7_9).
