# Supplementary material for: Earlier connectivity and specialization predict longitudinal changes in phonological and semantic specialization in 7- to 9-year-old children
Source: Imaging Neurosci (Camb). 2025 Oct 17;3:IMAG.a.940. doi: 10.1162/IMAG.a.940 (PMC12534709; doi:10.1162/IMAG.a.940)
Supplement: Supplementary Material [file IMAG.a.940_supp.pdf]

Supplementary Table 1 Comparison of average beta estimate (n=32) for phonological and semantic specialization within opIFG and trIFG (1 and 2), pSTG and pMTG (3 and 4), at both age 7 and age 9.

Age - 7		opIFG	trIFG	Paired t-test (p-value)
1	Phonological Specialization	8.88 (4.98)	- 11.92(6.24)	t (31) = 20.19, p < 0.001
2	Semantic Specialization	- 8.88 (4.98)	11.92 (6.24)	t (31) = -20.19, p < 0.001
		pSTG	pMTG	
3	Phonological Specialization	12.44 (6.17)	-15.90 (9.88)	t (31) = -15.88, p < 0.001
4	Semantic Specialization	- 12.44 (6.17)	15.90 (9.88)	t (31) = 15.88, p < 0.001
Age - 9		opIFG	trIFG	
1	Phonological Specialization	9.16 (3.77)	- 10.58 (5.06)	t (31) = 21.89, p < 0.001
2	Semantic Specialization	-9.16 (3.77)	10.58 (5.06)	t (31) = -21.89, p < 0.001
		pSTG	pMTG	
3	Phonological Specialization	11.37 (4.69)	- 11.11 (8.25)	t (31) = -13.54, p < 0.001
4	Semantic Specialization	-11.37 (4.69)	11.11 (8.25)	t (31) = 13.54, p < 0.001
